# Lack of Scientific Evidence for the Use of Gestrinone in the Treatment of Lipedema: A Systematic Review

**DOI:** 10.7759/cureus.97213

**Published:** 2025-11-19

**Authors:** Alexandre C Amato, Juliana S Amato, Daniel Benitti

**Affiliations:** 1 Department of Vascular Surgery, Amato - Instituto de Medicina Avançada, São Paulo, BRA; 2 Department of Gynecology, Amato - Instituto de Medicina Avançada, São Paulo, BRA; 3 Department of Vascular and Endovascular Surgery, Medical Valens Center, São Paulo, BRA

**Keywords:** evidence-based medicine, gestrinone, hormonal treatment, hormones, lipedema, off-label use, systematic review, treatment

## Abstract

Lipedema is a chronic, progressive disorder marked by the abnormal accumulation of subcutaneous adipose tissue, predominantly in the lower body and almost exclusively affecting women. In recent years, the off-label use of gestrinone - a synthetic steroid with androgenic, antiprogestogenic, and weak estrogenic activity, originally approved only for endometriosis - has gained attention as a potential therapy for lipedema, particularly in the form of subcutaneous implants. This systematic review aimed to assess the efficacy and safety of gestrinone for this indication.

A systematic literature search was conducted in PubMed, MEDLINE, Cochrane Library, and LILACS; clinical trial registries (ClinicalTrials.gov and Brazilian Registry of Clinical Trials (ReBEC)); as well as national and international clinical guidelines and expert consensus documents published up to July 30, 2025, following PRISMA guidelines. Eligible studies included randomized trials, observational studies, systematic reviews, case series, and clinical guidelines. Study selection, data extraction, and quality assessment were performed independently by two reviewers, with a third resolving discrepancies. The search identified nine records across all databases, registries, and other sources. After removing one duplicate, eight unique records were screened. All four records from indexed databases underwent full-text assessment. After applying inclusion/exclusion criteria, no studies - randomized, observational, or otherwise - were identified that evaluated the use of gestrinone for lipedema. Likewise, no ongoing clinical trials were found. Clinical guidelines and position statements from professional societies and patient associations uniformly advise against the off-label prescription of gestrinone for lipedema, citing the absence of scientific evidence.

There is no scientific basis for the use of gestrinone in the management of lipedema. Healthcare providers should rely on evidence-based treatments, including compression therapy, tailored physical exercise, nutritional counseling, and psychological support and restrict hormonal interventions to ethically approved research protocols.

## Introduction and background

Lipedema is a chronic and progressive condition that affects almost exclusively women, characterized by the abnormal and symmetrical accumulation of adipose tissue, primarily in the lower extremities, sparing the feet [1,2]. This condition was first described in 1940 by Allen and Hines at the Mayo Clinic [3], and, despite its significant prevalence - estimated at around 12.3% of the female population [4] - it has remained underdiagnosed and poorly understood for decades. Although the hormonal influence on lipedema remains hypothetical, several authors have proposed that it may be a hormone-driven disorder, possibly associated with an imbalance of estrogen receptors - a higher proportion of ERβ in adipose tissue - and relative progesterone resistance, potentially contributing to inflammation, abnormal fat deposition, and fibrosis. Lipedema should not be confused with hyperlipidemia (a metabolic disorder involving elevated blood lipids) or with obesity, as its pathophysiology, clinical presentation, and management differ substantially.

The classic symptoms of lipedema include pain, pressure sensitivity, easy bruising, and body disproportion [5]. Lipedema is often confused with obesity or lymphedema, which contributes to incorrect diagnoses and inadequate treatments [6]. The pathophysiology of lipedema is not yet fully understood, but studies suggest the involvement of genetic, hormonal, and inflammatory factors [7]. Although lipedema may coexist with obesity, it is a distinct pathological entity with unique clinical and histological features.

In the search for new therapeutic options for lipedema, the use of gestrinone, a synthetic steroid with androgenic, weak anti-progestogenic, and estrogenic properties, has been carried out in Brazil, driven partly by hypotheses that its hormonal modulation might influence lipedema pathophysiology. Gestrinone was originally developed and approved for the oral treatment of endometriosis [8]. However, in recent years, there has been an increase in the off-label use of gestrinone for various conditions, including lipedema, primarily in the form of subcutaneous implants - a practice that has raised concerns in the medical community due to the lack of pharmacokinetic, safety, and efficacy data for this route of administration.

According to the Brazilian Society of Endocrinology and Metabolism (SBEM), there are no studies on the safety and efficacy of gestrinone for parenteral use, particularly through implants [9]. The SBEM further emphasizes that gestrinone has anabolic actions and is on the list of prohibited substances in sports, according to the World Anti-Doping Agency (WADA) [10].

The current evidence-based treatment for lipedema includes compression therapy, adapted physical exercises, complex decongestive physical therapy, nutritional guidance, psychological support and, in selected cases, liposuction with lymphatic preservation [2,7,11]. These therapeutic approaches are recommended by international guidelines and expert consensus.

This study aims to conduct a systematic review of the scientific literature to evaluate the evidence on the efficacy and safety of gestrinone in the treatment of lipedema and to compare it with currently recommended evidence-based treatments.

## Review

A systematic literature review was conducted following the PRISMA (Preferred Reporting Items for Systematic Reviews and Meta-Analyses) guidelines [12]. The search was carried out in the following electronic databases: PubMed, MEDLINE, Cochrane Library, and LILACS. Studies published up to July 30, 2025, were included, with no restrictions on language. The complete search strategy is available in Appendix 1.

The search terms used included: "lipedema" OR "lipoedema" AND "gestrinone" OR "gestrinona" OR "hormonal therapy" OR "hormone treatment" OR "progestin" OR "progestogen" OR "androgen." Additionally, the clinical trial registries ClinicalTrials.gov and ReBEC (Brazilian Registry of Clinical Trials) were consulted to identify ongoing studies. Google Scholar was also systematically searched to identify potential grey literature and non-indexed publications. The search terms used were: "gestrinone" AND ("lipedema" OR "lipoedema").

Clinical guidelines and consensus documents on lipedema published by national and international medical societies were also analyzed, including the Brazilian Society of Angiology and Vascular Surgery (SBACV), the Brazilian Association of Lipedema, the International Society of Lymphology, the German Society of Phlebology, and the British Lymphology Society. Gestrinone is not approved or marketed in the United States and, therefore, is not considered a legally available drug by the Food and Drug Administration (FDA) [13].

The inclusion criteria were: (1) randomized clinical trials, (2) observational studies, (3) systematic reviews and meta-analyses, (4) case series and case reports specifically addressing the use of gestrinone in the treatment of lipedema, and (5) clinical guidelines and consensus documents on the treatment of lipedema.

The exclusion criteria were: (1) studies that did not specifically address lipedema, (2) studies on the use of gestrinone for conditions other than lipedema, (3) opinion articles without scientific references, and (4) advertising or promotional materials.

Two independent reviewers conducted the study selection, data extraction, and assessment of methodological quality. A third reviewer was consulted in case of disagreement. Had eligible studies been identified, the methodological quality of randomized clinical trials would have been assessed using the Jadad scale, and that of observational studies, using the Newcastle-Ottawa scale. Clinical guidelines would be evaluated using the AGREE II instrument (Appraisal of Guidelines for Research and Evaluation). Since no studies met the inclusion criteria, quality assessment was not performed. The study was approved by the Ethics Committee of the institution under protocol no. 01042025.

**Figure 1:**
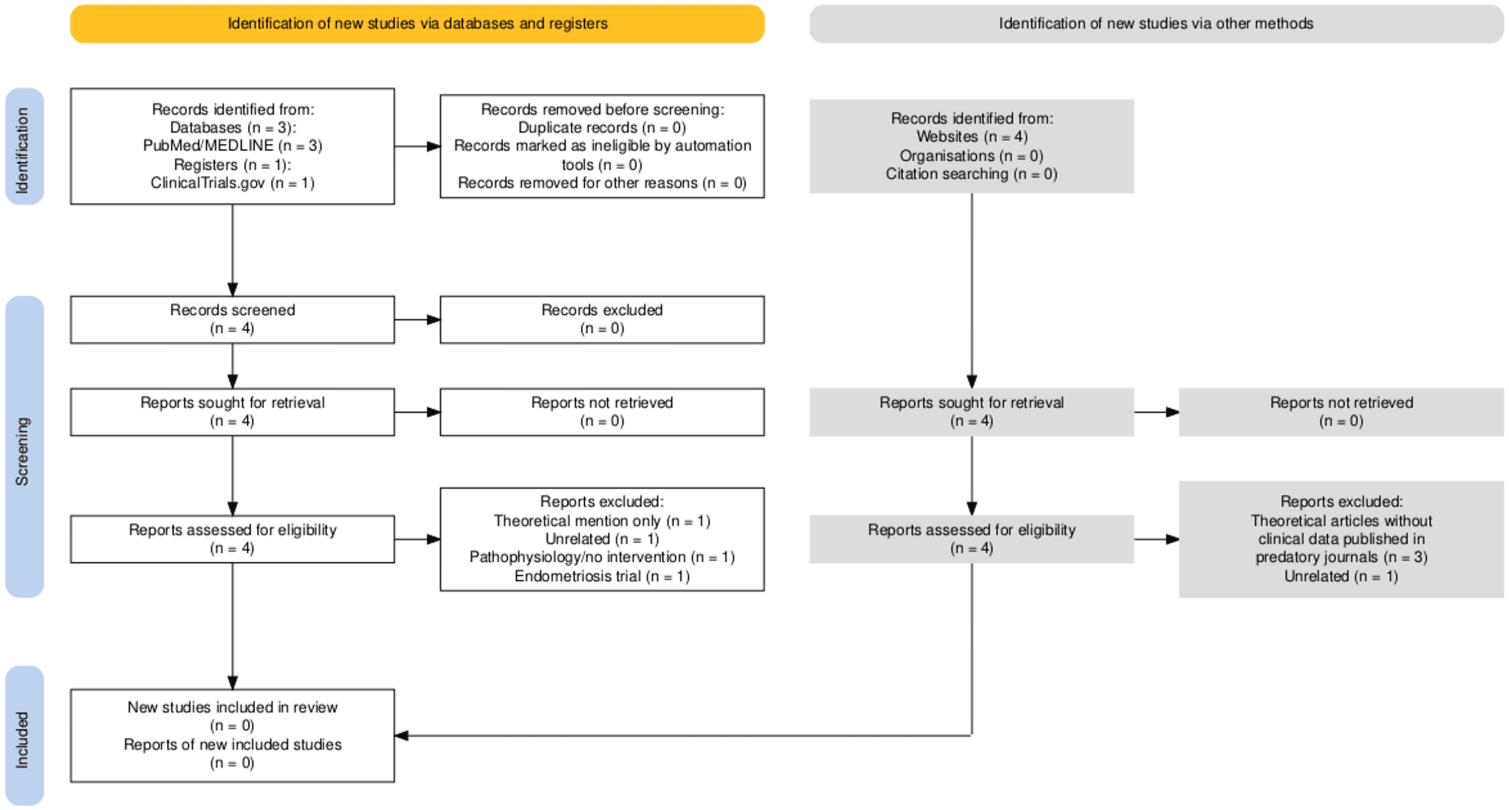
PRISMA Flow Diagram Showing the Study Selection Process The systematic search identified nine records across all sources (PubMed, ClinicalTrials.gov, and Google Scholar). After the removal of one duplicate, eight unique records were screened. The Google Scholar records were excluded during initial screening because they did not contain clinical data, addressed unrelated conditions, or consisted of theoretical or opinion-based material. Four full-text reports from indexed databases were assessed for eligibility; all were excluded because they did not study the use of gestrinone in patients with lipedema. No randomized trials, observational studies, case series, or case reports met the inclusion criteria. Consequently, zero studies were included in the final review.

The systematic search identified nine records across all databases, registries, and other sources (Figure 1). After the removal of one duplicate, eight unique records were screened. Four records from indexed databases and registries (PubMed/MEDLINE and ClinicalTrials.gov) proceeded to full-text assessment, while the remaining records retrieved from Google Scholar were excluded during initial screening because they did not present clinical data, addressed unrelated conditions, or consisted solely of theoretical or opinion-based material. Ultimately, no study met the inclusion criteria, and no evidence evaluating the use of gestrinone for lipedema was identified.

Four full-text reports from indexed databases underwent full eligibility assessment, but all were excluded because they did not investigate the use of gestrinone for lipedema or lacked clinical data. No records identified through database searches, registries, or grey literature fulfilled the inclusion criteria. Consequently, no studies were available for qualitative or quantitative synthesis.

After applying the inclusion and exclusion criteria, no studies were identified that specifically evaluated the use of gestrinone in the treatment of lipedema. No randomized clinical trials, observational studies, systematic reviews, or case reports investigating the efficacy or safety of gestrinone in the treatment of lipedema were found. Searches in clinical trial registry platforms also did not identify any ongoing studies on the subject. A PRISMA flow diagram depicting the study selection process is presented in Figure 1.

The analysis of clinical guidelines and consensus on the treatment of lipedema did not reveal any recommendations for the use of gestrinone or other progestogens in the treatment of this condition. The Brazilian Consensus on Lipedema, published by the SBACV in 2025, does not mention gestrinone among the recommended treatments [6].

The Brazilian Association of Lipedema published an official position in 2024, against the use of gestrinone in the treatment of lipedema, citing the "absence of scientific evidence to justify its use for this purpose" [7]. This position emphasizes that "any consideration of the use of gestrinone as a treatment for lipedema should be framed within an experimental context, under the supervision of a rigorous scientific research protocol approved by a medical ethics committee" [7].

In a similar position, the SBEM expressed opposition to the use of gestrinone implants, stating that "it does not recognize gestrinone implants as a therapeutic option," and that "there is no quality scientific evidence regarding the efficacy and safety of gestrinone implants" [9].

Additionally, the use of gestrinone in hormonal implants does not have approval from the National Health Surveillance Agency (ANVISA) for the treatment of lipedema [14]. ANVISA had two registrations for oral gestrinone for endometriosis (Dimetrose and Nometriós), which were canceled in 2012 and 2014. Currently, there are no products with gestrinone registered with ANVISA, and there are no studies demonstrating safety and efficacy via parenteral administration [15,16].

Recommended treatments for lipedema based on scientific evidence

The clinical guidelines and consensus analyzed recommend the following therapeutic approaches for lipedema, based on scientific evidence: (i) Compression Therapy, use of compression stockings or bandages to improve circulation and reduce discomfort [1,2,5,6]; (ii) Complex Decongestive Therapy (CDT), combination of manual lymphatic drainage, skin care, exercises, and compression [2,5,6]; (iii) Adapted Physical Exercises, low-impact activities such as swimming, water aerobics, and walking to maintain mobility and manage weight [2,5,6]; (iv) Nutritional Guidance, anti-inflammatory and balanced diets for weight control and reduction of systemic inflammation [2,5,6]; (v) Psychological Support, approaches to address body image issues and the psychosocial impact of the disease [2,5,6]; (vi) Liposuction With Lymphatic Preservation may be considered as an experimental adjunct therapy in carefully selected cases demonstrating severe functional impairment and inadequate response to at least one year of conservative management. However, given lipedema's underlying autoimmune and inflammatory mechanisms, comprehensive medium- to long-term surveillance is imperative - including objective assessment of metabolic parameters, inflammatory markers, and potential disease progression - rather than relying exclusively on patient-reported symptomatic outcomes. Notably, approximately 51% of post-surgical patients require continued conservative therapy, underscoring that surgical intervention does not eliminate the need for ongoing multimodal management [2,5,6,17].

The Brazilian Lipedema Consensus, using the Delphi methodology and published in 2025, emphasizes the need for a multidisciplinary approach, including physicians, physiotherapists, nutritionists, and mental health professionals [6]. This document highlights that "multidisciplinary care is essential for comprehensive management" of lipedema.

According to international guidelines, such as the Standard of Care for Lipedema in the United States [5] and the S2k Guideline Lipedema from Germany [18], conservative treatment should be the first-line approach for lipedema, with surgical interventions considered only in selected cases, and after adequate attempts at conservative treatment [17].

Discussion

This systematic review did not identify any scientific evidence supporting the use of gestrinone in the treatment of lipedema. The complete absence of clinical trials, observational studies, or even case reports on the subject is concerning, particularly considering the increasing use of this substance in clinical practice.

Gestrinone is a synthetic steroid with androgenic, weak antiprogestogenic, and estrogenic properties, approved only for the treatment of endometriosis via the oral route [8,19,20]. Its efficacy in treating endometriosis has been demonstrated in controlled clinical studies, but even for this approved indication, the scientific literature is limited and relatively old [8].

The off-label use of medications is a common practice in medicine and can be justified when there is scientific evidence supporting such use, even if it is not approved by the regulatory agency [20,21]. However, in the case of gestrinone for the treatment of lipedema, there is no evidence to justify this practice. Furthermore, the use of gestrinone in the form of subcutaneous implants completely lacks studies on pharmacokinetics, safety, and efficacy [9].

Gestrinone has recognized anabolic properties, which led to its inclusion on the list of prohibited substances by WADA [10]. Studies show that the use of anabolic substances is associated with various adverse effects, including liver changes, cardiovascular disorders, psychiatric alterations, and virilization in women [22]. The growing promotion of gestrinone for lipedema appears to be driven by marketing through social media and aesthetic medicine channels, capitalizing on patient desperation for effective treatments rather than robust clinical evidence.

The increasing use of gestrinone in the treatment of lipedema appears to be more related to marketing issues and the promise of quick aesthetic results than to solid scientific evidence. The phenomenon of gestrinone prescription for lipedema in Brazil raises important questions about medical practice patterns in countries with different regulatory frameworks. The availability of compounding pharmacies and the cultural acceptance of off-label hormonal therapies may contribute to this practice, highlighting the need for stronger regulatory oversight and physician education. This practice raises important ethical concerns, especially considering that patients with lipedema often face frustrating journeys in search of proper diagnosis and treatment, making them potentially more vulnerable to proposals for unproven treatments [23].

Lipedema is a complex condition that requires an evidence-based, multidisciplinary approach. The treatments recommended in current clinical guidelines and consensus - such as compression therapy, adapted physical exercises, CDT, nutritional guidance, and psychological support - are supported by scientific literature, with studies demonstrating their efficacy in improving symptoms and the quality of life of patients [24,25].

Liposuction with lymphatic preservation, when indicated in selected cases, also has growing, but still limited, scientific evidence of medium- and long-term benefits [17].

It is important to emphasize that, although there are hypotheses about the role of female hormones in the pathophysiology of lipedema, these are still preliminary and do not justify hormonal interventions as treatment [26,27]. Some studies have observed exacerbation of lipedema symptoms during periods of hormonal fluctuation, such as puberty, pregnancy, and menopause, but there are no studies demonstrating the benefits of hormonal manipulation in the treatment of this condition [27,28]. The prescription of compounded, non-FDA-approved hormonal therapies may expose patients to significant safety risks compared to approved alternatives [29]. International guidelines emphasize the importance of evidence-based care in lipedema management [30], and any future investigation into hormonal therapies should be conducted under rigorous research protocols, with appropriate ethical oversight [31]. The need for continued high-quality research to advance understanding and develop effective treatments for this challenging condition remains paramount [32].

Limitations

This systematic review has inherent limitations. Despite our comprehensive search strategy across multiple databases and clinical trial registries, we cannot entirely exclude the possibility of unpublished studies, ongoing unreported investigations, or gray literature that may exist outside indexed databases. The review protocol was not pre-registered in PROSPERO or similar platforms, although the risk of reporting bias is minimal, given the finding of zero eligible studies. Additionally, while no language restrictions were applied, potential language bias cannot be fully excluded, and not all international medical societies were directly consulted. However, the complete absence of any peer-reviewed evidence, registered clinical trials, or endorsement from professional medical societies strongly reinforces our primary conclusion. The lack of even preliminary case reports or observational data is particularly noteworthy, given the reported clinical use of this intervention.

Implications for future research

Given the preliminary nature of hormonal hypotheses in lipedema pathophysiology, any future investigation into endocrine modulation, including gestrinone, should adhere to rigorous scientific standards. Well-designed studies would require: (1) randomized, placebo-controlled trials with adequate sample sizes; (2) comprehensive pharmacokinetic studies, particularly for subcutaneous implant formulations; (3) long-term safety monitoring for hepatic, cardiovascular, and endocrine adverse effects; (4) standardized outcome measures, including objective assessment of limb volume, pain scores, and quality of life; and (5) mandatory ethical approval and informed consent processes that clearly communicate the experimental nature of such interventions. Until such evidence emerges, the prescription of gestrinone for lipedema remains scientifically unjustified.

## Conclusions

This systematic review highlights the complete absence of scientific evidence supporting the use of gestrinone in the management of lipedema. No clinical trials, observational studies, or systematic reviews were found addressing this treatment, and current clinical guidelines and expert consensus do not endorse its use. Evidence-based approaches for lipedema include compression therapy, adapted physical exercise, CDT, nutritional guidance, psychological support, and, in selected cases, liposuction with lymphatic preservation. These methods are recognized as safe and effective components of multidisciplinary care. Prescribing gestrinone - particularly in the form of hormonal implants - has no scientific foundation and may expose patients to unnecessary risks. Medical societies, such as the Brazilian Association of Lipedema and the SBEM, have already positioned themselves against this practice. Healthcare professionals are encouraged to adhere to evidence-based protocols and to avoid unproven therapies.

Future research exploring the hormonal aspects of lipedema should follow rigorous scientific standards, with ethical approval and informed consent. This review underscores the urgent need for rigorous, ethically approved trials before any hormonal therapy can be considered for lipedema. Promoting high-quality studies is essential to deepen understanding of this condition and to guide the development of safe, effective, and scientifically validated treatments. Until such evidence emerges, clinical practice should remain grounded in the principles of non-maleficence and evidence-based medicine.

## Appendices

Appendix 1: Complete search strategy

This supplementary material provides complete documentation of the systematic search strategy conducted following PRISMA 2020 guidelines to identify all available evidence on gestrinone treatment for lipedema. Searches were performed across multiple electronic databases, clinical trial registries, and grey literature sources, from inception to July 30, 2025, with no language restrictions.

Electronic Database Searches

Table 1 presents the detailed search strategy and results for each electronic database consulted.

**Table 1:** Database Search Strategy and Results

Database	Search Date	Search String	Filters	Results	Relevant Records
PubMed/MEDLINE	July 28, 2025	("lipedema"[All Fields] OR "lipoedema"[All Fields]) AND ("gestrinone"[All Fields] OR "gestrinona"[All Fields] OR "hormonal therapy"[All Fields] OR "hormone treatment"[All Fields] OR "progestin"[All Fields] OR "progestogen"[All Fields] OR "androgen"[All Fields])	None	3	3
Cochrane Library	July 28, 2025	(lipedema OR lipoedema) AND (gestrinone OR hormonal therapy)	None	0	0
LILACS	July 28, 2025	(tw:(lipedema OR lipoedema)) AND (tw:(gestrinone OR gestrinona OR "hormonal therapy"))	None	0	0

Clinical Trial Registry Searches

Table 2 summarizes searches conducted in clinical trial registries to identify ongoing or completed studies.

**Table 2:** Clinical Trial Registry Search Results

Registry	Search Date	Search Terms	Results	Relevant Trials
ClinicalTrials.gov	July 28, 2025	Condition: lipedema; Intervention: gestrinone	0	0
ClinicalTrials.gov	July 28, 2025	Condition: lipedema; Intervention: hormonal therapy	0	0
ReBEC	July 28, 2025	Lipedema AND gestrinone	0	0

Grey Literature and Additional Sources

Table 3 documents searches of clinical guidelines, position statements, and other grey literature sources.

**Table 3:** Grey Literature Search Results

Source Type	Organization/Source	Documents Reviewed	Gestrinone Mentioned?	Recommendation
Clinical Guidelines	SBACV (Brazil)	Brazilian Consensus 2025	No	Not recommended
Position Statement	Brazilian Lipedema Association	Official Position 2024	Yes	Explicitly against use
Position Statement	SBEM (Brazil)	Official Position 2021	Yes	Against implants
Website	Google Scholar	5 records (1 duplicate removed)	Yes (4 records)	All excluded (theoretical/predatory)

Search Methodology Notes

Search dates: July 25-28, 2025; search team: Alexandre C. Amato (lead), Daniel Benitti (verification), Juliana S. Amato (quality check). Total records identified: nine (four from databases/registries; five from Google Scholar). Final result: zero eligible studies. Protocol deviations: PROSPERO pre-registration was not completed due to the focused nature of the research question and zero preliminary findings. Embase and Web of Science were not searched, given comprehensive coverage by PubMed/MEDLINE and consistently zero findings.
